# Left Atrial Appendage Closure with Amplatzer Cardiac Plug in Nonvalvular Atrial Fibrillation: Safety and Long-Term Outcome

**DOI:** 10.5935/abc.20170167

**Published:** 2017-12

**Authors:** Marcio José Montenegro da Costa, Esmeralci Ferreira, Edgard Freitas Quintella, Bernardo Amorim, Alexandre Fuchs, Ricardo Zajdenverg, Hugo Sabino, Denilson Campos de Albuquerque

**Affiliations:** 1 Universidade do Estado do Rio de Janeiro (UERJ); - Rio de Janeiro, RJ - Brazil; 2 Instituto Estadual de Cardiologia Aloysio de Castro (IECAC); - Rio de Janeiro, RJ - Brazil

**Keywords:** Atrial Fibrillation, Atrial Appendage, Thrombosis / prevention & control, Vascular Closure Devices

## Abstract

**Background:**

Atrial fibrillation (AF) is a cardiac arrhythmia with high risk for thromboembolic events, specially stroke.

**Objective:**

To assess the safety of left atrial appendage closure (LAAC) with the Amplatzer Cardiac Plug for the prevention of thromboembolic events in patients with nonvalvular AF.

**Methods:**

This study included 15 patients with nonvalvular AF referred for LAAC, 6 older than 75 years (mean age, 69.4 ± 9.3 years; 60% of the male sex).

**Results:**

The mean CHADS_2_ score was 3.4 ± 0.1, and mean CHA_2_DS_2_VASc , 4.8 ± 1.8, evidencing a high risk for thromboembolic events. All patients had a HAS-BLED score > 3 (mean, 4.5 ± 1.2) with a high risk for major bleeding within 1 year. The device was successfully implanted in all patients, with correct positioning in the first attempt in most of them (n = 11; 73.3%).

**Conclusion:**

There was no periprocedural complication, such as device migration, pericardial tamponade, vascular complications and major bleeding. All patients had an uneventful in-hospital course, being discharged in 2 days. The echocardiographic assessments at 6 and 12 months showed neither device migration, nor thrombus formation, nor peridevice leak. On clinical assessment at 12 months, no patient had thromboembolic events or bleeding related to the device or risk factors. In this small series, LAAC with Amplatzer Cardiac Plug proved to be safe, with high procedural success rate and favorable outcome at the 12-month follow-up.

## Introduction

Atrial fibrillation (AF) is a cardiac arrhythmia of clinical relevance, because it significantly increases the risk for thromboembolic events, which not only worsen the quality of life but decrease life expectancy as well. That arrhythmia favors the formation of thrombi in the left atrial appendage (LAA), and any presentation of AF correlates with a twice higher occurrence of stroke than that in the general population. In up to 90% of the cases of AF, thrombi are found inside the LAA,^1^ particularly in patients with nonvalvular AF (NVAF), those without rheumatic mitral valve disease, valvular prosthesis or previous mitral valvuloplasty.^2^ In the presence of valvulopathy, thrombi usually occur in the left atrium. Approximately 20% of strokes are estimated to be associated with AF, frequently leading to death or disability.^3,4^

Regarding the prevention of thromboembolic events, it is important to individualize each patient’s risk. The score initially used to estimate the occurrence of those events is CHADS_2_.^5^ But, because it underestimates the risk in patients at lower risk, CHA_2_DS_2_VASc has been used in individuals with a CHADS_2_ score of 2.^6^ In individuals at high risk, those with a CHADS_2_ or CHA_2_DS_2_VASc score > 2, the prevention of NVAF-related thromboembolic events is usually pharmacological, by use of anticoagulants (AC).

In that population, hemorrhagic events are estimated by using the HAS-BLED score.^7^ For patients at high risk of bleeding or with contraindication for AC, left atrial appendage closure (LAAC) is an alternative strategy for the mechanical prevention of thromboembolic events.

The primary objective of this study was to assess the safety of LAAC performed with the Amplatzer Cardiac Plug™ (ACP) device by analyzing the immediate and in-hospital safety outcomes. The secondary objective was to assess that strategy to prevent major cardiovascular events in late follow-up.

## Methods

This was a retrospective, longitudinal, cohort study with prospective data collection of patients with NVAF submitted to LAAC with ACP, from November 2010 to March 2015, at the Instituto Estadual de Cardiologia Aloysio de Castro. The study was approved by the Research Ethics Committee of that institution (nº 049018). All patients provided written informed consent.

### Selection of patients

The patients included in this study met the following three criteria:

- NVAF with high risk for thromboembolic events:
  a.1) CHADS > 2 or CHADS = 2 and CHA_2_DS_2_VASc > 2;
  a.2) or NVAF with previous history of stroke, transient ischemic attacks (TIA), or peripheral embolism;
- Objective evidence of limitation to the use of AC with:
  b.1) previous history of hemorrhagic stroke or major bleeding;
  b.2) or high risk for bleeding with HAS-BLED ≥ 3;
  b.3) or unstable response to the use of AC, defined as less than 60% of prothrombin time (PT) readings within the limits of the therapeutic range (INR ≤ 2.0 or INR ≥ 3.0) in the past 12 months;
- Provided written informed consent.

Patients with the following conditions were excluded from this study:

- Severe consumptive disease with life expectancy shorter than 1 year, or clinical contraindication to the intervention on the initial assessment;
- Echocardiographic evidence of pathologies that could lead to thromboembolic events or cerebral ischemia, such as intracardiac thrombi, valvulopathies, ulcerated aortic atherosclerotic plaque, or significant obstruction of the carotid or vertebral arteries;
- Unsuitable anatomy for the procedure, with LAA waist diameter on echocardiography smaller than 12 mm or larger than 28 mm.

### Device

**Figure 1:**
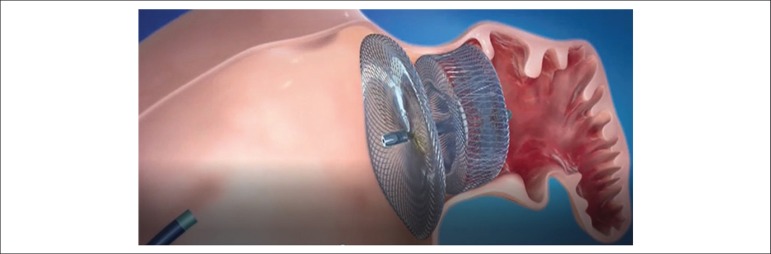
Amplatzer Cardiac Plug (image provided by St. Jude Medical Inc.).

The ACP device (Figure 1) has been previously described in the *Arquivos Brasileiros de Cardiologia* on the occasion of our initial experience.^8^

### Study protocol

During hospitalization, the following safety outcomes were considered: death, stroke, device migration, pericardial tamponade, and vascular and hemorrhagic complications.

During inpatient follow-up, safety outcomes, such as device embolization, pericardial effusion and major bleedings were compiled. During inpatient and outpatient follow-ups, the occurrence of efficacy outcomes, such as adverse clinical events (death, cardiovascular death, stroke, TIA and thromboembolism), was assessed. Telephone contact was performed on days 30, 60, 180 and 365. The occurrence of thrombosis, device migration and peridevice leak was assessed by use of transesophageal echocardiography on days 180 and 365.

### Procedure

**Figure 2:**
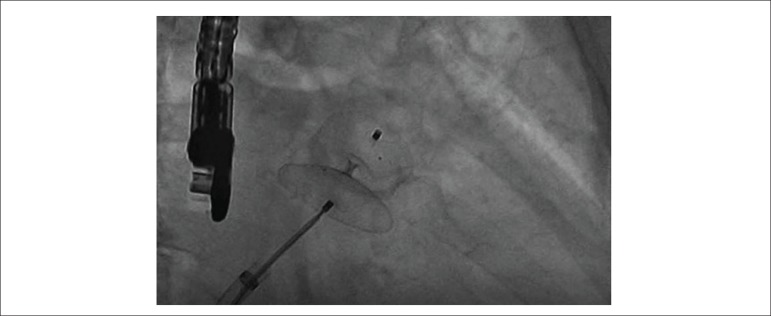
Angiography (caudal RAO): the aspect of “smashed tire” characterizes the proper size of the prosthesis.

Coumarins were suspended 4 days before the procedure. Prophylactic heparinization was implemented with low-molecular-weight heparins. All procedures were performed under general anesthesia. Vascular access was performed via right femoral vein puncture, with initial placement of a 5F or 6F introducer, for the posterior introduction of a 7F or 8F Mullins sheath. Transseptal puncture was performed under two-dimensional transesophageal echocardiography on bicaval view, allowing perfect visualization of the fossa ovalis in the interatrial septum, where puncture with transseptal needle was preferentially performed in the postero-inferior position, which, in addition to being far from the aorta, facilitates selective LAA catheterization. After transseptal puncture, unfractionated heparin, at the dose of 70-100 IU/kg, was injected, maintaining activated coagulation time (ACT) longer than 250 seconds during the procedure. Then, the Mullins sheath was inserted through the septum and a rigid 1.5 cm guidewire with flexible tip was positioned in the left superior pulmonary vein for further introduction of a 10F to 13F delivery catheter. With the delivery catheter placed inside the LAA, the ACP device was delivered through over the deployment wire until exteriorization of the lobe release catheter beyond the circumflex artery, which is perfectly visible under transesophageal echocardiography, so that the ACP device would stabilize and acquire the aspect of a smashed tire, showing its adequate size. With the lobe properly positioned in the landing zone, the proximal disk was unfolded to cover the LAA entrance. Good device positioning was assessed under echocardiography and fluoroscopy, confirming the LAAC (Figure 2 and videos 1 and 2). Finally, the delivery cable was removed, leaving the LAA occluder in its proper place. Then, the presence of residual flow in the interatrial septum was assessed, being the femoral vein sheath removed.

**Video 1:** Angiography (caudal RAO): implantation with proper disk concavity, separation between disk and lobe, and proper alignment of the device. Note the absence of flow in the left atrial appendage and patent left superior pulmonary vein.

**Video 2:** Angiography (caudal RAO): Minnesota maneuver, pushing and drawing of the device to ensure adequate anchoring, immediately before its release.

Immediately after the procedure, the AC were suspended, and dual antiplatelet therapy (DAPT) initiated with acetylsalicylic acid (ASA - 200 mg/day) and clopidogrel (attack dose of 300 mg, and maintenance of 75 mg/day) for 6 months. Before hospital discharge, the presence of complications was assessed with transesophageal echocardiography.

## Results

### Baseline characteristics

Eighteen patients with indication for LAAC were referred for ACP device implantation. On transesophageal echocardiography assessment, 3 patients showed thrombi inside the LAA: 2 on the preprocedural assessment, and 1 in the 4-day period between warfarin suspension and procedure performance. After exclusion of those 3 patients, 15 were left to be assessed. Table 1 shows their clinical characteristics. It is worth noting that all patients were hypertensive and had permanent AF. There was high association with coronary artery disease, myocardial revascularization (n = 11; 73.3%) and previous stroke or TIA (n = 9; 60%).

**Table 1:** Characteristics of the population studied

Characteristics	Continuous variables (mean ± SD)
Age (years)	69.4 ± 9.3
CHADS_2_ score	3.4 ± 0.1
CHA_2_DS_2_-VASc score	4.8 ± 1.8
HAS-BLED score	4.5 ± 1.2
**Characteristics**	**Categorical variables - n (%)**
Age > 65 years	11 (73.3%)
Age > 75 years	6 (40.0%)
Male sex	9 (60.0%)
Permanent atrial fibrillation	15 (100.0%)
Persistent atrial fibrillation	-
Heart failure	6 (40.0%)
Arterial hypertension	15 (100.0%)
Diabetes mellitus	5 (33.3%)
Previous stroke/TIA	9 (60.0%)
Carotid disease	-
Coronary disease	11 (73.3%)
Acute myocardial infarction	8 (53.3%)
PCA	8 (53.3%)
CABG	3 (20.0%)
Peripheral embolism	1 (0.07%)
Unstable INR	11 (73.3%)

TIA: transient ischemic attack; SD: standard deviation; PCA: percutaneous coronary angioplasty; CABG: coronary artery bypass grafting; INR: international normalized ratio.

One patient with indication for coronary angioplasty had an episode of hematuria when using the association of DAPT and warfarin. Three days after coronary stent implantation, the patient underwent LAAC, and hematuria resolved after suspending the AC.

### Risk scores

The mean CHADS_2_ score was 3.4 ± 0.1, and the mean CHA_2_DS_2_VASc, 4.8 ± 1.8, evidencing the high risk for thromboembolic events in this cohort (Table 2).

**Table 2:** CHADS_2_ and CHA_2_DS_2_VASc scores

Patients	CHADS_2_		CHA_2_DS_2_VASc	
	score	Stroke risk 100 patients/year (95%CI)	score	Stroke risk 100 patients/year (95%CI)
1	4	8.5 (6.3 - 8.1)	6	3.6 (0.4 - 12.6)
2	5	12.5 (8.2 - 17.5)	8	11.1 (0.3 - 48.3)
3	4	8.5 (6.3 - 8.1)	6	3.6 (0.4 - 12.6)
4	5	12.5 (8.2 - 17.5)	7	8.0 (1.0 - 26.0)
5	2	4.0 (3.1 - 5.1)	3	3.9 (1.7 - 7.3)
6	2	4.0 (3.1 - 5.1)	4	1.9 (0.5 - 4.9)
7	4	8.5 (6.3 - 8.1)	5	3.2 (0.7 - 9.0)
8	5	12.5 (8.2 - 17.5)	7	8.0 (1.0 - 26.0)
9	4	8.5 (6.3 - 8.1)	5	3.2 (0.7 - 9.0)
10	5	12.5 (8.2 - 17.5)	6	3.6 (0.4 - 12.6)
11	2	4.0 (3.1 - 5.1)	3	3.9 (1.7 - 7.3)
12	2	4.0 (3.1 - 5.1)	4	1.9 (0.5 - 4.9)
13	3	5.9 (4.6 - 7.3)	4	1.9 (0.5 - 4.9)
14	2	4.0 (3.1 - 5.1)	3	3.9 (1.7 - 7.3)
15	2	4.0 (3.1 - 5.1)	2	1.6 (0.3 - 4.7)

All patients had a HAS-BLED score > 3 (mean of 4.5 ± 1.2), showing the high risk for major bleedings in this population (Table 3). Many patients (n = 11; 73.3%) had an unstable INR, which was the major factor in the HAS-BLED score of this population, evidencing the difficulty of maintaining therapeutic levels of coumarins in the real world.

**Table 3:** HAS-BLED score

Patients	HAS-BLED	
	score	Bleeding risk 100 patients/year
1	4	8.70
2	4	8.70
3	6	> 12.5
4	6	> 12.5
5	3	3.74
6	3	3.74
7	5	12.5
8	6	> 12.5
9	5	12.5
10	6	> 12.5
11	4	8.70
12	4	8.70
13	5	12.5
14	3	3.74
15	3	3.74

Because of the inclusion criteria, all patients had NVAF and high risk for bleeding. In addition, the major indication for LAAC was history of major bleedings (n = 11; 73% - the digestive tract being the major site) and INR instability (73%). Other factors that could influence on the choice of that strategy are shown in Table 4.

**Table 4:** Indications for LAAC

Indications for LAAC	n (%)
High risk of bleeding	15 (100.0)
History of major bleeding	11 (73.3)
Unstable INR	11 (73.3)
**Other situations observed**	
History of stroke using AC	9 (60.0)
Coronary disease and stent	8 (53.3)
History of minor bleeding	3 (20.0)
Liver or kidney disease	2 (13.3)

LAAC: left atrial appendage occluder; INR: international normalized ratio; AC: anticoagulants.

### Percutaneous intervention

Device implantation was successful in all patients. In most of them (n = 11; 73.3%), proper positioning was achieved in the first attempt, while 4 patients required other attempts. The access was always via transseptal puncture. No patient required exchanging the device for another of different size. When required, coronary intervention with stent implantation was performed before LAAC. Two patients had Bezold-Jarisch reflex with bradycardia and hypotension during the delivery catheter introduction, which, in such cases, had the largest caliber (13F) because larger devices were implanted (28 and 30 mm). Table 5 lists the sizes of the devices implanted.

**Table 5:** Characteristics of the device implanted

ACP size	n (%)
16 mm	-
18 mm	1 (0.07)
20 mm	2 (13.3)
22 mm	-
24 mm	3 (20.0)
26 mm	4 (26.7)
28 mm	4 (26.7)
30 mm	1 (0.07)

ACP: Amplatzer Cardiac Plug™.

One patient showed hypokinetic left ventricular lateral wall at the end of the procedure, although the controls immediately after the procedure (transesophageal echocardiogram and coronary angiography) evidenced no compression of the circumflex artery by the device.

### Follow-up

the patients had a favorable in-hospital outcome, 2 days being the mean hospital length of stay due exclusively to the procedure. In the in-hospital echocardiographic follow-up, neither residual flow in the interatrial septum nor thrombi on the device was observed.

One patient had cerebral arteriovenous malformation with no possibility of surgical treatment, which contraindicated any type of AC. Before LAAC, all other patients were on any combination of antithrombotic medications, which always included warfarin. The new oral AC were not used by that population and were not available at our institution. The use of antithrombotic medication after LAAC was restricted to the indications associated with the presence of coronary disease, and no patient used AC after LAAC (Table 6).

**Table 6:** Antithrombotic drugs

	Pre-intervention n (%)	Follow-up n (%)
ASA		8 (53.3)
ASA + coumarins	7 (46.7)	
ASA + clopidogrel		3 (20.0)
ASA + clopidogrel + coumarins	4 (26.7)	
Coumarins	3 (20.0)	
None	1 (6.6)	4 (26.7)

ASA: acetylsalicylic acid.

The echocardiographic assessments at 6 and 12 months showed no complication, such as device migration, thrombi or peridevice leaks. On clinical assessment at 12 months, no patient had thromboembolic events or bleeding, showing a favorable clinical course.

### Discussion

Three clinical trials assessing the efficacy of LAAC as compared to warfarin have been published, none, however, with the ACP device. The PROTECT-AF Trial (*Percutaneous closure of the left atrial appendage versus warfarin therapy for prevention of stroke in patients with atrial fibrillation trial*) has selected 800 patients in 59 centers in the United States and Europe, testing the non-inferiority hypothesis of the Watchman device as compared to warfarin, in random grouping 2:1 (device:control), with transesophageal echocardiography follow-up (45 days, 6 months and 1 year).^9^ The primary efficacy outcomes observed were stroke, cardiovascular or unexplained death, and systemic embolism. The safety outcomes were device embolization, pericardial effusion and major bleedings. The results showed a 32% risk reduction with the device. The use of coumarins was suspended by 87% of the patients. However, there were more episodes of pericardial effusion in the device group, probably due to the learning curve of the technique. The study has concluded that the device proved not to be inferior to coumarins, with better survival free from events and stroke. In that study, a CHADS score around 2 and the use of warfarin in the first 45 days after implantation could have interfered with the short-term results.

The PREVAIL Trial (*Prospective randomized evaluation of the Watchman left atrial appendage closure device in patients with atrial fibrillation versus long-term warfarin therapy*) has included 461 patients at the proportion of 2:1 (device:control) and has also assessed the non-inferiority hypothesis, but in a population with a higher CHADS_2_ score.^10^ The primary outcomes were similar to those observed in the PROTECT-AF: stroke, cardiovascular or unexplained death, and systemic embolism. That study has shown that, even in patients at higher risk, the device can be safely implanted. But the pre-specified criteria of non-inferiority were not met, although the event rates were within the expected range. The authors concluded that the Watchman device is a safe alternative to anticoagulation therapy to prevent thromboembolic events in patients with NVAF.

The EWOLUTION was a prospective, multicenter, nonrandomized cohort study that assessed more than 1000 patients at high risk for stroke (mean CHA_2_DS_2_-VASc: 4.5 ± 1.6) and moderate to high risk for bleeding (mean HAS-BLED: 2.3 ± 1.2).^11^ Almost half of the patients had a previous history of TIA or ischemic or hemorrhagic stroke. The success rate of the Watchman device implantation was 98.5%, much higher than that reported in the PROTECT-AF Trial (90.9%). Serious complications related to the procedure were observed in only 8.7% of the interventions, a 2.7% lower rate than that of the PROTECT-AF Trial. The success rate of the Watchman device implantation was high, with low pre-procedure risk, although that cohort of patients had higher comorbidities and high risk of stroke and bleedings. For the authors, this could be attributed to the improvement in the implantation technique, which significantly reduced the complications that limited the clinical benefits of that therapy.^11^

In our series of 15 patients, LAAC with ACP device proved to be safe in patients with NVAF, showing neither thromboembolic nor hemorrhagic events in the 12-month follow-up, and a high success rate of implantation (n = 15; 100%). The major indications for the intervention were the high risk for bleeding and INR instability, both in 73.3% of the patients. Because many patients (n = 11; 73.3%) had associated coronary disease, requiring antiplatelet aggregation, the risk for bleeding increased in that population, although the age group of our sample (69.0 ± 9.0 years) was lower than those in other studies, such as the PROTECT-AF Trial (72.0 ± 9.0 years) and the PREVAIL Trial (74.0 ± 7.0 years).^10,11^

The use of warfarin was very difficult in that population, with 11 patients (73.3%) unable to remain in the therapeutic range, with high INR instability, and 9 patients (60.0%) reporting stroke despite the use of warfarin. Some studies have suggested that only more educated patients could realize the risk of bleeding, the possibility of interaction with other drugs and the need for continuous therapeutic monitoring.^12^ Thus, the rates of undertreatment or non-treatment with coumarins in high-risk patients varies in some series between 20% and 80%, although only 15% of the patients with AF have any contraindication for AC.^13-15^

None of our patients used the new oral AC, which were not available in the public healthcare system. In this series, warfarin was parsimoniously used, because 73.3% of the patients had established coronary artery disease.^16^ Almost half of the patients in this cohort (n = 7; 46.7%) used AC and DAPT, while 4 patients (26.7%) used the association of AC and ASA. Under such situations, either the patient already had AF and required a coronary intervention with stent implantation, or was submitted to stent implantation and developed AF later. In both situations, when using the conventional stent, AC and DAPT are usually associated for 1 month only, clopidogrel being then suspended, and only the AC and ASA maintained. When a drug-eluting stent is used, DAPT should persist for 6 to 12 months,^17^ which increases the risk for bleeding with the association of DAPT and AC.

In this unfavorable scenario, some studies have tested three possibilities: triple therapy (TT) with the association of DAPT and AC; dual therapy (DT) with the association of AC and ASA or clopidogrel, and DAPT alone.^18^ Regarding major adverse cardiac events, there was no difference between those three groups. When assessing stroke and embolic events outside the central nervous system, the prevalence of events was higher in the DAPT group. The TT and DT groups did not differ regarding stent thrombosis and embolic events, although the TT group showed a higher rate of bleedings.^17^ Recently, the prospective and randomized WOEST study, with almost 600 patients, showed no superiority of TT over DT to prevent embolic or major adverse cardiac events, confirming the higher rate of bleeding with TT.^19^ Thus, LAAC seems more advantageous to patients with coronary artery disease associated with NVAF, to which there is no definition about the ideal antithrombotic therapy. One should consider the risks of stent thrombosis and embolic events due to AF, as well the risk for bleedings due to drug association.^5^

In this study, all patients had a HAS-BLED score greater than 3 (mean of 4.5 ± 1.2), with a highly elevated risk of bleeding. In a risk x benefit assessment, that might not be a reason to suspend the AC, because the patients with high risk for embolic events and bleeding are probably those that benefit most from the use of AC. However, they might also be the ones that benefit most from a local intervention, such as LAAC.^20^ In addition, 11 patients in this series (73.3%) had high INR instability with the use of AC, and, in such cases, a prevention strategy without the AC is highly desirable. Finally, in the physical and mental assessment of a subgroup of 547 patients from the PROTECT-AF Trial, an improvement in the quality of life was demonstrated, favoring the patients who underwent LAAC as compared to those treated with warfarin.^20^

Device implantation was successful in all patients, and that might be attributed to the small sample size (15 patients). But we observed some details that could interfere in those results. First, it is important to define the anatomical shape of the LAA, and in those with more than one lobe, try to observe if their bifurcation site is close to the ostium, which hinders the device implantation. Second, and maybe even more important, to define the size of the device to be implanted. After performing the LAA measurements recommended, the device used should be 20% larger, which hinders its positioning, but ensures perfect occlusion, decreasing the chance of its migration. We believe that such measures can prevent the device exchange for another of different size. In the PROTECT-AF, PREVAIL and EWOLUTION trials, the number of prostheses used per patient were 1.6, 1.5 and 1.1, respectively,^9-11^ but all those studies used the Watchman device, while, in our patients, the ACP device was used. Currently, there is the second generation of the ACP device, the Amulet occlude device, used in the same way, but with a lower peridevice leak rate. In a series of 59 consecutive patients of a single center, the Amulet occluder device has shown performance similar to that of the ACP in the periprocedural and short-term outcomes, but with significant reduction in peridevice leaks.^21^

A study of 1998 assessed the antithrombotic regimens after coronary stent implantations.^22^ Currently some studies are attempting to discover the best therapy for the patient submitted to LAAC. But, in the absence of specific studies with LAAC devices, that result was transferred to ACP, and DAPT was instituted after the implantation. In our series, almost all patients (n = 14; 93.3%) were on warfarin before the procedure, which was suspended 4 days before ACP implantation. Although DAPT is programmed for 6 months in the outpatient follow-up, only 20% of the patients use it.

In the EWOLUTION study,^11^ where one fourth of the patients received no preprocedural AC and 6% continued without it after the procedure, there were several therapeutic possibilities for the follow-up, such as DAPT (59.6%), single antiplatelet aggregation (7.1%), new AC (11.1%), and warfarin alone (15.6%). The groups did not differ regarding embolic events, but there was a slightly lower rate of bleedings with the use of new AC.^11^

Two studies have investigated the use of adjuvant drug therapy with the Watchman device,^11,23^ but only one study assessed the use of ACP.^24^ In that study, with more than 1000 patients, compiling results from 22 centers, the annual rate of systemic thromboembolism was 2.3% after LAAC, representing a 59% risk reduction. The annual rate of major bleedings was 2.1%, representing a 61% risk reduction. Monotherapy with ASA or no medication was performed after LAAC, resulting in a reduction in hemorrhagic events. Considering that warfarin was the drug used for the comparison, all those studies are somehow outdated, because the currently used new oral AC have superior results.^11^

The European guideline recommends LAAC for patients who have life-threatening hemorrhage with no reversible cause, who cannot use anticoagulation (class IIb, level of evidence B).^25^

## Conclusions

LAAC is a therapeutic alternative for patients with NVAF and difficulties with anticoagulation, either due to contraindications or history of severe adverse events when using anticoagulation, or even for patients at high risk for bleeding, prohibitive HAS-BLED score, because it proved to be safe considering its low complication rates and high procedural success rate.
